# The complete mitochondrial genome of *Lutraria maxima* Jonas (Veneroida: Mactridae)

**DOI:** 10.1080/23802359.2017.1390403

**Published:** 2017-10-14

**Authors:** Shengping Zhong, Yanfei Zhao, Xianfeng Wang, Zhifei Song, Qing Zhang

**Affiliations:** Key Laboratory of Marine Biotechnology, Guangxi Institute of Oceanology, Beihai, China

**Keywords:** Mitochondrial genome, *Lutraria maxima*, bivalves

## Abstract

The snout otter clam *Lutraria maxima* Jonas (Veneroida: Mactridae) is an economically important species in the marine bivalves fishery and aquaculture in China. However, the genetic information of this species remains unavailable. In this study, we report the complete mitochondrial genome sequence of *L. maxima*. The mitogenome has 17,082 base pairs (63.9% A + T content) and made up of a total of 37 genes (13 protein-coding, 22 transfer RNAs and two ribosomal RNAs), and a 1045 bp non-coding control region. The presence of a gene coding for ATPase subunit 8 was also noted. However, as expected in Mactridae, the gene arrangements showed variations with that of the related species. This study adds to the repository of available mitogenomes of various Mactridae and will provide useful genetic information for future fishery management and aquaculture development of Mactridae.

The genus *Lutraria* is medium-sized marine bivalve known as snout otter clam, which is an ecologically and commercially important species in Asia (Gan et al. 2016). The snout otter clam, being commercial fisher and increasingly aquafarming in Southeast Asia and southeast coastal region in China, has become an increasingly important aquaculture species in countries such as Vietnam and China (Su et al. 2009; Luca and Nam 2011). In spite of its commercial importance, adequate genetic information about this species and the genus is still missing. Here, we report the complete mitochondrial genome sequence of *Lutraria maxima*, which will be an important genetic resource to assist in molecular identification of this species and help improve phylogenetic and population genetic studies of the genus *Lutraria*.

A tissue sample of *L. maxima* was collected from Guangxi province, China (Beihai, 21°25′59.47″N, 109°16′43.34″E), and the whole body specimen (#GR0108) were deposited at Marine biological Herbarium, Guangxi Institute of Oceanology, Beihai, China. The total genomic DNA was extracted from the muscle of the specimens using an SQ Tissue DNA Kit (OMEGA, Guangzhou, China) following the manufacturer’s protocol. DNA libraries (450 bp insert) were constructed with the TruSeq NanoTM kit (Illumina, San Diego, CA) and were sequenced (2 × 150 bp paired-end) using HiSeq platform at Novogene Company, China. Mitogenome assembly was performed by MITObim (Hahn et al. 2013). The complete mitogenome of *L. rhynchaena* (GenBank accession number: NC_023384) was chosen as the initial reference sequence for MITObim assembly. Gene annotation was performed by MITOS (Bernt et al. 2013).

The complete mitogenome of *L. maxima* was found to be 17,082 bp in length (GenBank accession number: MF784266), consisting of the usual set of 13 protein-coding, 22 tRNA and two rRNA genes and a putative control region. The overall base composition of the mitogenome was estimated to be A 22.7%, T 41.2%, C 10.9% and G 25.1%, with a high A + T content of 63.9%, which is similar, but slightly different from *L. rhynchaena*. The gene order in *L. maxima* is highly similar to that found in *L. rhynchaena* except in the presence of ATPase subunit 8 (ATP8), which is likely to be due to the use of a different annotation pipeline for the gene identification (Gan et al. 2016). The result of the phylogenetic tree of 13 species (including other 12 species from order Veneroida in NCBI) also supported the close relationship between *L. maxima* and *L. rhynchaena* (Figure 1), as they shared the same branch node with the highest bootstrap value. All protein-coding genes were found to use the initiation codon ATG except for NAD6, ATP8, ATP6, NAD3 and NAD5 genes, where ATT served as the initiation codon. COX1 and COX3 terminated with an incomplete stop codon T, which is thought to be completed with the addition of 3’ adenine residues to the mRNA (Ojala et al. 1981). Among the 37 genes, three intergenic were identified. The complete mitochondrial genome sequence of *L. maxima* adds to the number of sequenced mitogenomes within the family Mactridae, which will contribute to further phylogenetic and comparative mitogenome studies of the family Mactridae and related families.

**Figure 1.**
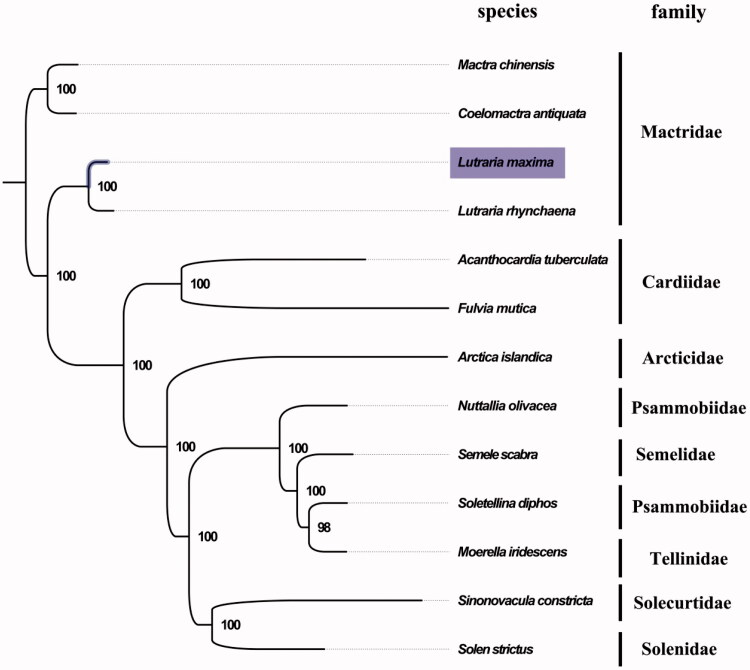
Phylogenetic tree of 13 species in order Veneroida. The complete mitogenomes is downloaded from GenBank and the phylogenic tree is constructed by a maximum-likelihood method with 100 bootstrap replicates. The bootstrap values were labelled at each branch nodes. The gene’s accession number for tree construction is listed as follows: *Mactra chinensis* (NC_025510), *Coelomactra antiquata* (NC_021375), *L. rhynchaena* (NC_023384), *Acanthocardia tuberculata* (NC_008452), *Fulvia mutica* (NC_022194), *Arctica islandica* (NC_022709), *Nuttallia olivacea* (NC_018373), *Semele scabra* (NC_018374), *Soletellina diphos* (NC_018372), *Moerella iridescens* (NC_018371), *Sinonovacula constricta* (NC_011075), and *Solen strictus* (NC_017616).
